# Early and Mid-Term Ultrasound Response to Guselkumab in Psoriatic Arthritis: A Real-World Cohort from a Single Tertiary Rheumatology Center

**DOI:** 10.3390/jcm15031196

**Published:** 2026-02-03

**Authors:** Filippo Messina, Massimo Caimi, Linda Lucchetti, Marco Bonifacio, Emanuele Fiorino, Alessandro Conforti

**Affiliations:** 1 ASL Roma 4, 00053 Civitavecchia, Italy; filippo.messina@aslroma4.it (F.M.); massimo.caimi@aslroma4.it (M.C.); lida.lucchetti@aslroma4.it (L.L.); emanuele.fiorino@aslroma4.it (E.F.); 2 Fisioterpia Medica, 00144 Rome, Italy; marcobonifacio1969@gmail.com

**Keywords:** psoriatic arthritis, guselkumab, power doppler ultrasound, GLOESS, global synovitis score, enthesitis, OMERACT scoring

## Abstract

**Objective:** To quantify early and mid-term changes in ultrasound-detected synovitis and enthesitis after initiating guselkumab in psoriatic arthritis (PsA) and to contextualize imaging responses alongside clinical outcomes. **Methods:** We conducted a retrospective single-center cohort study of consecutive CASPAR-classified adults (*n* = 20) initiating guselkumab 100 mg (week 0/week 4, then q8w; q4w intensification per routine practice). Power Doppler ultrasound (PDUS) followed EULAR–OMERACT standards at baseline (T0), Month 3 (T3), and Month 6 (T6). The primary endpoint was within-patient change in 24-joint GLOESS. Secondary endpoints included OMERACT entheseal scores (activity-only; activity + structure), DAPSA states, PASI, and 6-month persistence. Within-patient changes were assessed using Wilcoxon signed-rank tests (two-sided). **Results:** All 20 patients completed T6. GLOESS decreased from T0 to T3 (mean Δ −4.05 ± 2.78; *p* = 0.0312) and to T6 (mean Δ −5.70 ± 4.05; *p* = 0.0001). Component summaries showed a numerically larger early decrease in synovial PD signal than in grayscale synovial hypertrophy (descriptive). Enthesitis scores improved: OMERACT activity-only median Δ −2.0 at T3 (*p* = 0.0313) and −3.5 at T6 (*p* = 0.016); activity + structure median Δ −1.5 at T3 (*p* = 0.0412) and −3.0 at T6 (*p* = 0.031). The estimated structural component (OMERACT-1 minus OMERACT-2) was similar across visits (descriptive), indicating improvements driven predominantly by inflammatory signal suppression rather than detectable changes in structural lesions over 6 months. Among patients with baseline PD-positive enthesitis (*n* = 7), PD negativity (OMERACT-2 = 0) occurred in 2/7 (28.6%) by T6. Clinical domains (DAPSA, PASI) improved in parallel, and 6-month persistence was high. **Conclusions:** In routine care, guselkumab was associated with a significant improvement in PD-inclusive ultrasound synovitis scores by 3 months, which deepened by 6 months, alongside an improvement in entheseal activity measures. Over 6 months, entheseal structural burden appeared stable; these findings should be considered hypothesis-generating and warrant confirmation in prospective controlled studies.

## 1. Introduction

Psoriatic arthritis (PsA) is a chronic, immune-mediated spondyloarthritis with intrinsically multidomain expression across peripheral joints, entheses, skin, and nails. Patients manifest variable mixes of synovitis, enthesitis, dactylitis, and axial disease that complicate case finding, phenotyping, and longitudinal monitoring [1]. The legacy notion of PsA as benign has been refuted by contemporary registries showing destructive, progressive disease with quality-of-life decrements comparable to rheumatoid arthritis, alongside frequent cardiometabolic multimorbidity [1]. Diagnostic delay is clinically meaningful; even 6–12 months of delay is associated with greater long-term joint damage and sustained disability, underscoring the need for early recognition of active inflammation and timely initiation of effective therapy [1].

Synovitis and enthesitis are central drivers of pain, dysfunction, and structural outcomes in PsA. On ultrasound, synovitis is defined by hypoechoic synovial hypertrophy (SH) with or without power Doppler (PD) signal, while entheseal inflammation at fibro-cartilaginous insertions contributes to the characteristic combination of bone erosion and new bone formation across the spondyloarthritis spectrum [2,3]. The synovio-entheseal complex concept links mechanically induced inflammation at the enthesis to adjacent synovium, helping explain lesion co-localization and peri-/peritendinous patterns typical of PsA [3]. Clinically, enthesitis is difficult to detect and often under-ascertained: site tenderness has limited sensitivity and only fair agreement with imaging, whereas ultrasound can reveal subclinical activity at joints and entheses with prognostic and therapeutic implications [2,4].

Musculoskeletal ultrasound (MSUS) provides real-time visualization of SH (grayscale) and microvascular flow (PD), enabling sensitive detection of inflammatory activity beyond clinical examination. In early PsA, MSUS can identify substantial “silent” diseases; among 49 recent-onset, DMARD-naïve cases, 75.5% had subclinical synovitis at clinically inactive sites, and three-quarters of clinically oligoarticular presentations were reclassified as polyarticular based on ultrasound findings [5]. Such observations support imaging-anchored treat-to-target (T2T) strategies, particularly when clinical examination underestimates inflammatory burden.

PD is a proximate marker of vascularized inflammation in synovium and enthesis, but its sensitivity hinges on optimized techniques, including low pulse-repetition frequency and wall filter, high-frequency linear transducers, and minimal probe compression [3]. Methodological standardization by OMERACT, in collaboration with EULAR, has defined elementary lesions and semi-quantitative grades for synovitis and enthesitis, improving reproducibility and enabling patient-level composites such as the Global OMERACT–EULAR Synovitis Score (GLOESS) [2,3]. Reliability is robust: the OMERACT enthesitis definition retains hypoechogenicity, thickening, calcifications/enthesophytes, erosions, and PD at the insertion, and PD at the enthesis shows near-excellent reliability (prevalence-adjusted κ ~ 0.9) [2]. For small-joint synovitis, combined EULAR–OMERACT grading shows good intra-/inter-reader agreement for both grayscale and PD, and dorsal PD interrogation can improve discrimination compared with volar views [6]. Collectively, these standards position MSUS as an objective biomarker of current inflammatory activity and short-term treatment response, complementary to clinical indices and acute-phase reactants.

Biologically, PD mirrors dilated, hyperemic microvessels at the inflamed synovio-entheseal interface and typically declines rapidly with effective anti-inflammatory therapy, often preceding measurable change in grayscale SH or entheseal structural lesions. In the IL-17A inhibitor ULTIMATE trial, change in GLOESS separated from placebo by week 1 after secukinumab initiation and remained significantly lower at 12 weeks, while clinical secondary outcomes were concordant but less temporally sensitive than the PD-inclusive ultrasound score [7]. In routine care, imaging can also reveal residual inflammation despite apparent clinical control: in a cohort of 51 patients with DAPSA < 14, ultrasound detected active enthesitis in 19.6% and active synovitis in 15.7%, highlighting how PD-positive lesions can persist “at target” and may warrant therapy optimization [4].

Therapeutic options for PsA now span TNF inhibitors, IL-17 inhibitors, IL-12/23 inhibitors, selective IL-23p19 inhibitors, and Janus kinase inhibitors, selected according to domain involvement, comorbidities, and patient preference [1]. Within this landscape, guselkumab, an IL-23 p19-specific monoclonal antibody, has demonstrated robust, multidomain efficacy with favorable safety in phase 3 programs. In DISCOVER-1 (*n* = 381; ~30% prior TNFi), guselkumab 100 mg dosed q4w or at weeks 0/4 then q8w achieved week-24 ACR20 responses of 59% and 52% versus 22% for placebo [8]. In DISCOVER-2 (biologic-naïve; *n* = 739), responses were sustained or improved through week 52, with durable gains in dactylitis, enthesitis resolution, function, and quality of life, and low radiographic progression with infrequent serious infections [9]. Pooled analyses showed by week 52 that ~52–54% achieved DAPSA low disease activity, ~18% reached DAPSA remission, 31–36% met minimal disease activity (MDA), and ~13–14% attained very low disease activity (VLDA), with broadly similar efficacy across q4w and q8w regimens [10]. However, most trial evidence emphasizes clinical composites rather than standardized objective imaging correlates.

Standardized ultrasound correlates of guselkumab’s effect, especially short-term PDUS responses in real-world care, remain under-characterized. PDUS studies in PsA have focused mainly on IL-17 inhibition (e.g., ULTIMATE), demonstrating that PD-inclusive composites such as GLOESS are highly responsive to early therapeutic effects [7]. Addressing the IL-23 imaging gap matters for two pragmatic reasons. First, PD often decreases earlier than grayscale-defined SH and earlier than detectable change in entheseal structural lesions; therefore, proximal PD reductions may help contextualize continuation, intensification, or switching decisions at 3–6 months, common clinical decision points. Second, T2T implementation remains inconsistent in routine clinics; in one study, only 45% had DAPSA recorded, and ~51% achieved low disease activity/remission overall, with higher success among biologic-treated patients [11]. Embedding PDUS into follow-up may identify residual inflammatory activity in patients deemed “at target” clinically and support more consistent T2T behavior, including earlier dose-interval adjustments or mechanism changes [4,11].

Validated instruments exist to operationalize imaging targets. For joints, EULAR–OMERACT synovitis scoring permits patient-level GLOESS across a 24-joint-pair set, and reduced-joint approaches may approximate global scores while improving feasibility when key technical parameters, especially dorsal PD interrogation in small joints, are respected [6]. For entheses, OMERACT definitions allow parallel quantification of activity and damage to disentangle early anti-inflammatory effects from slower structural change [2]. Clinical entheseal indices capture different populations; ASAS-PerSpA data show that MEI and SPARCC identify more PsA patients with enthesitis than LEI or MASES, with meaningful sensitivity differences by subtype and site distribution [12]. Real-world persistence data suggest roughly two-thirds of PsA patients remain on TNF or IL-17 inhibitors at 6 months [13], but analogous IL-23p19 persistence contextualized by early PDUS dynamics is scarce.

Against this backdrop, we report, to our knowledge, the first single-center, real-life cohort integrating standardized, protocolized ultrasound endpoints with clinical outcomes under guselkumab at predefined early (3-month) and mid-term (6-month) checkpoints. Consecutive CASPAR-classified adults initiating guselkumab in routine care were assessed with high-frequency linear transducers and optimized Doppler settings, using EULAR–OMERACT-based scanning and scoring with consensus image reading. The primary endpoint was the change in PDUS-detected synovitis using patient-level GLOESS at 3 and 6 months [6,7], supported by secondary ultrasound and clinical outcomes to provide pragmatic effectiveness evidence. Collectively, converging PD-inclusive ultrasound and IL-23p19 inhibition supports a more precise, inflammation-anchored T2T approach in PsA [2,3,6,7,9,10,11,12].

## 2. Methods

### 2.1. Study Design and Setting

We performed a retrospective, real-life observational cohort study at the Rheumatology Center of ASL Roma 4, San Paolo Hospital (Civitavecchia, Italy). The observation window extended from December 2024 through October 2025. During this period, we reviewed clinical and ultrasound records for all consecutive adults with psoriatic arthritis (PsA) who initiated guselkumab as part of routine care. Data were abstracted from the center’s standardized PsA database and verified against electronic source documentation by two independent investigators to ensure completeness and accuracy. The rationale was to evaluate, for the first time in this setting, the short-term effectiveness of guselkumab on synovial and power Doppler inflammatory activity using an integrated clinical ultrasound approach. To our knowledge, this represents the first real-world assessment of early (3-month) and mid-term (6-month) ultrasound response to guselkumab.

### 2.2. Participants

Eligible patients were aged ≥ 18 years and met the CASPAR classification criteria for PsA. To capture an inflammatory phenotype likely to change with treatment, inclusion required at baseline: at least three tender and three swollen joints on 66/68 joint counts; active synovitis on power Doppler ultrasonography (PDUS) meeting a predefined positivity threshold; at least one clinically tender enthesis quantified using the SPARCC index; and availability of protocolized ultrasound assessments at baseline (T0), 3 months (T3), and 6 months (T6), together with complete clinical follow-up to T6. We excluded individuals with alternative rheumatologic diagnoses (including rheumatoid arthritis, non-psoriatic spondyloarthritis, or systemic connective-tissue diseases), those with missing or inadequate ultrasound data at any scheduled visit, patients with active infections, pregnancy, or recent malignancy, and records with suboptimal image quality in at least one planned assessment. The final sample comprised 20 consecutively treated patients who satisfied all criteria.

### 2.3. Treatment Exposure and Concomitant Therapies

All patients commenced guselkumab (Tremfya^®^, Janssen Biotech, Horsham, PA, USA) 100 mg subcutaneously according to the approved regimen (week 0, week 4, then every 8 weeks thereafter). In routine practice, intensification to every-4-week dosing (q4) was permitted for selected cases, typically after the T3 evaluation, at the treating physician’s discretion; timing and indication for intensification were recorded in the database. Concomitant conventional synthetic DMARDs (csDMARDs) and non-steroidal anti-inflammatory drugs (NSAIDs) were allowed, provided the dose had been stable for at least four weeks before guselkumab initiation and remained unchanged through T3 to avoid confounding early treatment effects. Importantly, no patient received systemic or local glucocorticoids through T3 in accordance with the protocol; after T3, treatment adjustments, including consideration of low-dose corticosteroids per clinical need, were allowed and documented. This strategy ensured that early ultrasound changes reflected guselkumab exposure rather than background medication shifts.

### 2.4. Outcomes and Operational Definitions

The primary objective was to quantify the short-term effectiveness of guselkumab on PDUS-detected synovitis, operationalized as a change in the EULAR-OMERACT Global Synovitis Score (GLOESS) at T3 and T6 relative to baseline. GLOESS was treated as a patient-level continuous outcome derived from standardized semi-quantitative joint grading (0–3) that integrates grayscale synovial hypertrophy and synovial Doppler signal at each joint.

Secondary objectives were prespecified to characterize broader PsA activity and treatment targets. First, we evaluated PDUS-based enthesitis according to OMERACT, reported in two complementary ways: “Definition 1,” a composite incorporating both activity and structural damage, and “Definition 2,” which isolates inflammatory activity. For each definition, we examined absolute values and change from baseline, and we explored the correspondence between PDUS enthesitis and clinical enthesitis measured by the SPARCC index. Second, we assessed joint disease activity using the Disease Activity Index for Psoriatic Arthritis (DAPSA), classifying patients at T3 and T6 into remission or low disease activity (LDA) categories. Third, we captured other PsA domains, specifically dactylitis (quantified by the Leeds Dactylitis Index, LDI) and skin involvement (Psoriasis Area and Severity Index, PASI). Fourth, we examined the proportion of patients achieving composite treatment targets at each follow-up-minimal disease activity (MDA), very low disease activity (VLDA), and DAPSA remission/LDA, reflecting multidomain control. Finally, we described 6-month treatment persistence and safety signals as captured by routine pharmacovigilance entries, including any adverse events (AEs), serious AEs, and withdrawals.

### 2.5. Ultrasound Acquisition and Scoring

All ultrasound procedures followed EULAR–OMERACT standardized definitions and scanning protocols for inflammatory arthritis. Examinations were performed on high-resolution platforms equipped with 12–18 MHz linear transducers (≥15 MHz for most assessments) with optimized musculoskeletal Doppler settings (pulse-repetition frequency 400–800 Hz; Doppler frequency 7–14 MHz). To minimize inter-machine variability, each patient was consistently assessed on the same device throughout follow-up.

Assessments were conducted by a dedicated team of three experienced physicians—two musculoskeletal sonographers and one rheumatologist, each with at least five years of formal experience in musculoskeletal ultrasound. The team engaged in regular inter-observer calibration sessions aligned with EULAR–OMERACT exercises. All images were digitally archived, and any discrepancies in scoring were resolved by consensus review to preserve inter-reader reliability across T0, T3, and T6.

For synovitis, we evaluated 24 bilateral joint pairs according to the EULAR–OMERACT definition. Each joint received a composite semi-quantitative grade from 0 to 3, informed by two components: hypoechoic synovial hypertrophy (grayscale) and the presence and extent of synovial PD signal. The patient-level GLOESS was calculated as the sum of composite grades across the 24 joint pairs, yielding a theoretical range of 0–144. This approach captures both structural and vascular features of synovitis and has demonstrated responsiveness to change in inflammatory arthritides, thereby supporting its use as the primary outcome.

For enthesitis, we examined six bilateral entheseal sites, the common extensor tendon, quadriceps tendon, proximal and distal patellar tendons, Achilles tendon, and plantar fascia, using OMERACT definitions. Each enthesis was scored with a semi-quantitative scale (0–3) for inflammatory abnormalities (e.g., PD signal, hypoechogenicity, thickening) and structural changes (enthesophytes, erosions). Two global patient-level enthesitis scores were derived: Definition 1 (activity plus structure), computed as PD 0–3 with the addition of a B-mode structural component 0–1 across sites (range 0–48), and Definition 2 (activity only), computed from PD 0–3 alone (range 0–36). These complementary indices allowed us to distinguish early anti-inflammatory effects (PD signal reduction) from entheseal structural lesions that are expected to remain relatively stable over short follow-up intervals.

### 2.6. Ultrasound Protocol

PDUS evaluation of synovitis and enthesitis was scheduled at T0, T3, and T6, and all clinical and imaging data were collected contemporaneously and analyzed retrospectively. For synovitis, the 24-joint bilateral set was scanned in standardized planes with machine presets optimized to detect low-velocity flow while minimizing artifact. For enthesitis, the six bilateral sites were imaged with careful attention to probe orientation and minimal compression to preserve PD sensitivity. Quality assurance procedures included routine phantom checks, saved presets, and periodic reviewer concordance exercises. Each patient’s serial examinations were performed on the same platform by the trained team to reduce technical heterogeneity over time.

### 2.7. Statistical Analysis

Continuous variables are summarized as mean (standard deviation, SD) and median (interquartile range, IQR); categorical variables are summarized as counts and percentages with denominators shown where missingness occurred. Within-patient changes from baseline (T0) to Month 3 (T3) and Month 6 (T6) were evaluated using two-sided Wilcoxon signed-rank tests. A two-sided *p*-value < 0.05 was considered statistically significant. Given the exploratory nature of this small retrospective cohort, no multiplicity adjustment was applied. Analyses were performed using IBM SPSS Version 31.

### 2.8. Ethics Approval

This study was reviewed and approved by the Comitato Etico Territoriale Lazio Area 3 (approval No. 7728; protocol code BIRRA; decision dated 10 July 2025). This study was conducted in accordance with the Declaration of Helsinki. Clinical and ultrasound (Power Doppler) data were processed in coded form and analyzed after de-identification, in compliance with GDPR (EU 2016/679) and Italian privacy legislation (D.Lgs. 10 August 2018 n. 101).

## 3. Results

We analyzed 20 consecutive PsA patients with standardized clinical and PDUS assessments at baseline (T0), Month 3 (T3), and Month 6 (T6); all patients completed follow-up through T6 (Female sex, *n*/N (%) = 16/20 (80.0%)). Baseline characteristics are summarized in Table 1, with denominators shown where routine-care documentation resulted in missingness for selected variables. Patient-level 24-joint GLOESS improved from T0 to T3 and to T6, with significant within-patient changes at both timepoints (T0 → T3 *p* = 0.0312; T0 → T6 *p* = 0.0001; Table 2). The interquartile range at T6 was slightly smaller than at baseline (IQR 7.75 vs. 8.25; descriptive). Component summaries showed a numerically larger early decrease in PD signal than in grayscale synovial hypertrophy (descriptive), consistent with earlier suppression of Doppler-detected inflammatory activity and more gradual change in B-mode synovial hypertrophy.

Among those with available history, a family history of PsA was present in 23.5% (4/17) and of psoriasis in 35.3% (6/17). Articular pattern data, where recorded, suggested a predominance of oligoarticular disease (73.3%, 11/15), with polyarticular involvement in 29.4% (5/17) of a separately captured denominator. Axial involvement was frequent (61.1%, 11/18). Enthesitis at baseline was common clinically (70.6%, 12/17), whereas dactylitis was less prevalent (17.6%, 3/17). Denominators vary due to missingness; percentages are therefore reported relative to *n* for each row.

**Figure 1:**
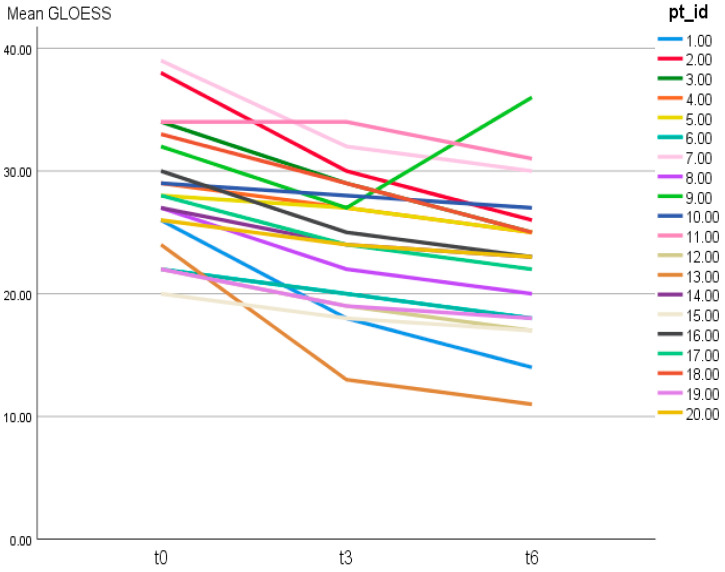
Individual trajectories of patient-level 24-joint GLOESS over time (T0, T3, T6). Each line represents one patient (pt_id = patient identifier).

**Figure 2:**
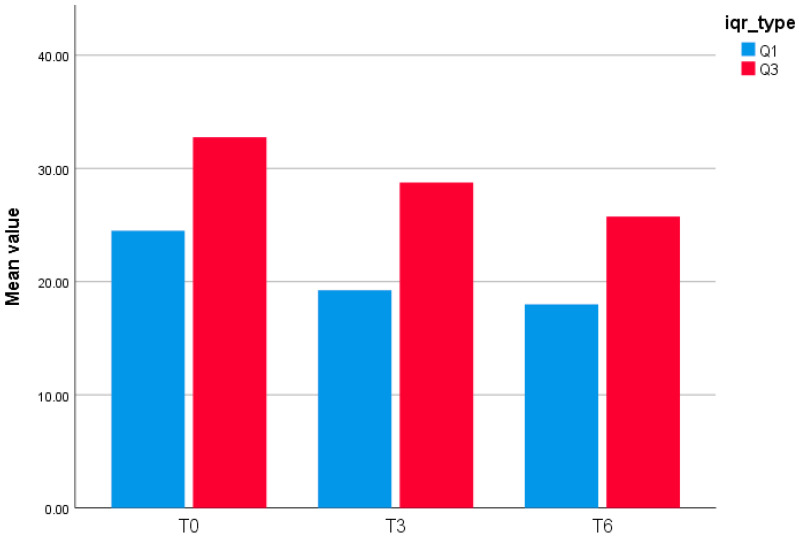
Interquartile Range (Q1–Q3) of GLOESS (24-joint) at T0, T3, and T6.

**Figure 3:**
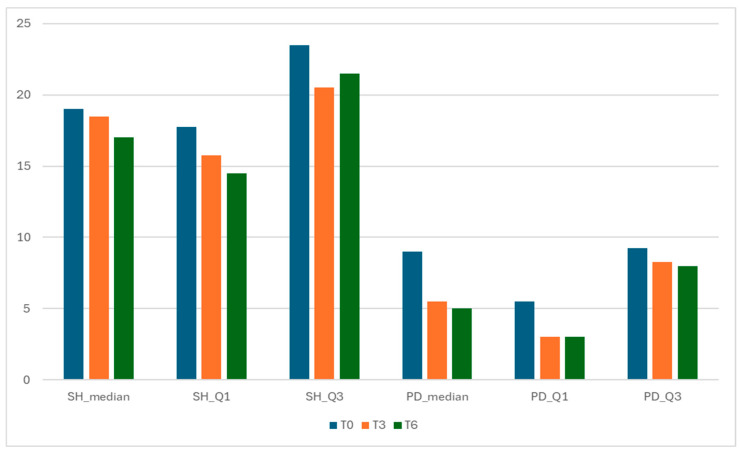
Components of GLOESS—Synovial Hypertrophy (SH) and Power Doppler (PD) distributions over time.

Patient-level GLOESS improved from T0 to T3 and T6, with significant within-patient change at both timepoints (T0 → T3 *p* = 0.0312; T0 → T6 *p* = 0.0001; Table 2). Individual trajectories and distributional shift show an early T0 → T3 drop, smaller gains thereafter, and narrowing variability by T6 (Figure 1 and Figure 2). The PD component declined earlier and more steeply than grayscale synovial hypertrophy, consistent with greater short-term responsiveness of PD signal compared with B-mode hypertrophy (descriptive) (Figure 3).

Individual trajectories of patient-level GLOESS across T0, T3, and T6 are shown in Figure 1, demonstrating a general downward shift over time (Figure 1).

This bar chart (Figure 2) displays the interquartile bounds (Q1 and Q3) of the GLOESS (Global Synovitis Score—24 joint) at three timepoints: baseline (T0), Month 3 (T3), and Month 6 (T6). At T0, the 25th percentile (Q1) was 24.5, and the 75th percentile (Q3) was 32.75. At T3, Q1 decreased to 19.25 and Q3 to 28.75, reflecting a shift in the distribution of synovitis scores. By T6, the interquartile range further narrowed, with Q1 at 18.0 and Q3 at 25.75. These results suggest a progressive reduction in synovitis burden over time, with compression of the score distribution.

Both structural (SH) and inflammatory (PD) components improved from T0 to T6, with a relatively larger drop in PD (vascular activity) (Figure 3). The relative magnitude of change was computed by comparing the absolute within-cohort change in component summary values from baseline (T0) to follow-up (T3 and T6). SH median decreased from 19.0 (IQR 17.75–23.50) at T0 to 18.5 (15.75–20.50) at T3 and 17.0 (14.50–21.50) at T6.

Within-patient analysis showed significant reductions in OMERACT entheseal scores at both checkpoints (Table 3). From baseline to Month 3, changes were significant for OMERACT-1 (activity + structure; median Δ −1.5; *p* = 0.0412) and OMERACT-2 (activity-only; median Δ −2.0; *p* = 0.0313). By Month 6, improvements deepened (OMERACT-1 median Δ −3.0; *p* = 0.031; OMERACT-2 median Δ −3.5; *p* = 0.016). Importantly, the estimated structural component—approximated by the difference between OMERACT-1 and OMERACT-2—remained similar across visits (descriptive), indicating that the observed improvement in the activity + structure construct was predominantly driven by suppression of inflammatory activity rather than detectable change in structural lesions over the 6-month interval (Table 3). To avoid overinterpretation, we report PD negativity specifically (OMERACT-2 = 0) rather than composite entheseal remission. Among patients with baseline PD-positive enthesitis (OMERACT-2 > 0; *n* = 7), PD negativity occurred in 2/7 (28.6%) by T6; structural lesions could still be present despite PD negativity. After the T3 assessment, guselkumab dosing was intensified to every 4 weeks (q4w) in patients due to persistent disease activity in routine care; the remaining patients continued q8w dosing. No patient discontinued guselkumab before T6.

## 4. Discussion

In this real-world cohort of 20 patients with psoriatic arthritis initiating guselkumab, we observed significant within-patient improvements in ultrasound-detected inflammatory activity at both joints and entheses over six months. Patient-level 24-joint GLOESS improved significantly from baseline to Month 3 and further to Month 6, with all patients completing follow-up. The interquartile range at Month 6 was slightly smaller than at baseline (descriptive), suggesting modest dispersion reduction without formal variance testing. Component summaries indicated a numerically larger early decline in synovial power Doppler (PD) signal compared with grayscale synovial hypertrophy, consistent with earlier suppression of Doppler-detected vascular activity followed by slower grayscale improvement. Enthesitis scores improved significantly under both OMERACT constructs, activity-only (OMERACT-2) and activity + structure (OMERACT-1). The difference between these constructs, used pragmatically as an estimate of structural burden, remained similar across visits (descriptive), implying that the composite improvement was primarily driven by reduced inflammatory activity rather than measurable structural change. Accordingly, changes over six months should be interpreted as suppression of inflammatory activity, with longer follow-up required to assess structural trajectories. Among seven patients with baseline PD-positive enthesitis, PD negativity (OMERACT-2 = 0) was achieved by two (28.6%) at Month 6; structural lesions could persist despite PD negativity.

Our findings align with the clinical efficacy established in randomized guselkumab trials (DISCOVER-1/2), which demonstrated robust improvement across joint and skin domains and durable disease control over 1–2 years [8,9,14]. The present imaging data complement those results by confirming that these clinical benefits are accompanied by objective suppression of vascular activity at the tissue level by three months and sustained improvement at six months. This temporal pattern parallels the ultrasound kinetics reported in the ULTIMATE study of IL-17A inhibition, where PD-inclusive GLOESS declined rapidly by week 12 with early separation from placebo [7,15]. Despite targeting distinct cytokine axes (IL-23 vs. IL-17A), both programs show a coherent pattern of PD signal suppression preceding slower grayscale change, suggesting a convergent anti-inflammatory trajectory under modern cytokine blockade.

Parallel improvement across clinical and ultrasound measures reinforces the translational significance of our observations. Declines in DAPSA, PASI, and VAS pain mirrored significant reductions in GLOESS and entheseal scores by Month 6, linking symptomatic improvement to tissue-level biology. Similar construct validity has been shown for EULAR-OMERACT ultrasound composites against clinical disease activity in other inflammatory arthritides [16], supporting the role of patient-level GLOESS as a feasible anchor outcome in both research and routine care. The full-cohort follow-up and paired design strengthen internal validity, and the observed six-month significance satisfies the reviewer’s emphasis on statistical robustness. These results are congruent with real-world evidence from CorEvitas, where persistent on-label guselkumab use correlated with composite disease activity and patient-reported improvement at six months [13]. Furthermore, favorable persistence relative to TNF inhibitors in claims analyses underscores the drug’s durable effectiveness [17].

The earlier reduction in PD signal relative to grayscale synovial hypertrophy observed here is biologically plausible, noting that this comparison is descriptive and was not tested with a formal component-by-time interaction. PD signal reflects microvascular hyperemia, which typically resolves rapidly once cytokine-driven endothelial activation subsides, whereas grayscale hypertrophy and structural enthesopathic features regress more slowly. This temporal hierarchy mirrors earlier findings under IL-17A inhibition [7,15] and is consistent with the observation that reductions in PD signal may occur before a detectable change in grayscale morphology. However, we note explicitly that this component sequence is descriptive and not supported by formal statistical testing of slope or variance differences. Similar caution applies to the observed IQR narrowing at Month 6, which, while suggestive of more uniform improvement, was not a prespecified endpoint. Both patterns are hypothesis-generating and require verification in larger prospective cohorts.

The enthesitis data warrant specific interpretation. Using standardized OMERACT definitions, we found that activity-only scores improved to a greater magnitude than the activity + structure composite over six months. This distinction underscores PD’s sensitivity as a dynamic biomarker of active inflammation, while structural elements such as erosions or enthesophytes remain stable in the short term. This aligns with OMERACT ultrasound task force findings on the reliability of PD at insertion sites [2,15,18,19,20] and supports PDUS as the most responsive modality for early biological change. Clinically, this distinction is critical: PD positivity at the enthesis identifies active vascular inflammation, whereas residual grayscale abnormalities often reflect chronicity. Adherence to EULAR–OMERACT scanning protocols [3] strengthens the reproducibility and interpretability of these results.

The relationship between clinical enthesitis indices and ultrasound findings deserves attention. Granados et al. (2023) demonstrated variable agreement among clinical indices such as SPARCC, MASES, MEI, and LEI, highlighting limitations in detecting biologically active enthesitis [12]. Our dual use of SPARCC for clinical and OMERACT-based PDUS for imaging captured both symptomatic and biological domains. The discordance observed, where PD resolution occurred despite residual tenderness, illustrates PDUS’s capacity to differentiate nociplastic or mechanical pain from true inflammation, particularly in PsA, where central sensitization can confound clinical scores. Agache et al. (2024) similarly found that up to 20% of PsA patients in clinical remission retained PD-positive lesions, emphasizing PDUS’s role in uncovering residual disease [4]. Such findings underscore that PDUS augments, rather than duplicates, clinical assessment by revealing the biological substrate of residual symptoms.

**Figure 4:**
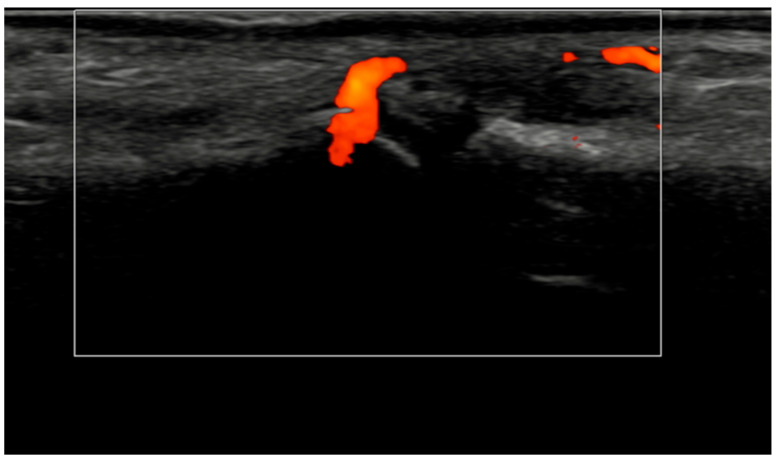
Longitudinal ultrasound image of the PIP III joint showing synovial hypertrophy (grade 2) and intra-articular power Doppler signal with distal capsular and entheseal extension (PD grade 2), corresponding to a combined EULAR–OMERACT synovitis grade = 2 (joint level).

**Figure 5:**
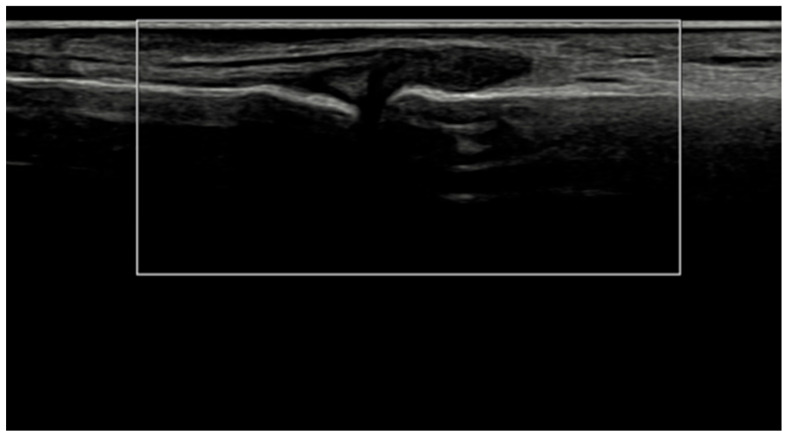
Longitudinal ultrasound image of the PIP II joint showing synovial hypertrophy (grayscale grade 2) without detectable intra-articular power Doppler signal (PD grade 0), corresponding to a combined EULAR–OMERACT synovitis grade = 2 (joint-level).

**Figure 6:**
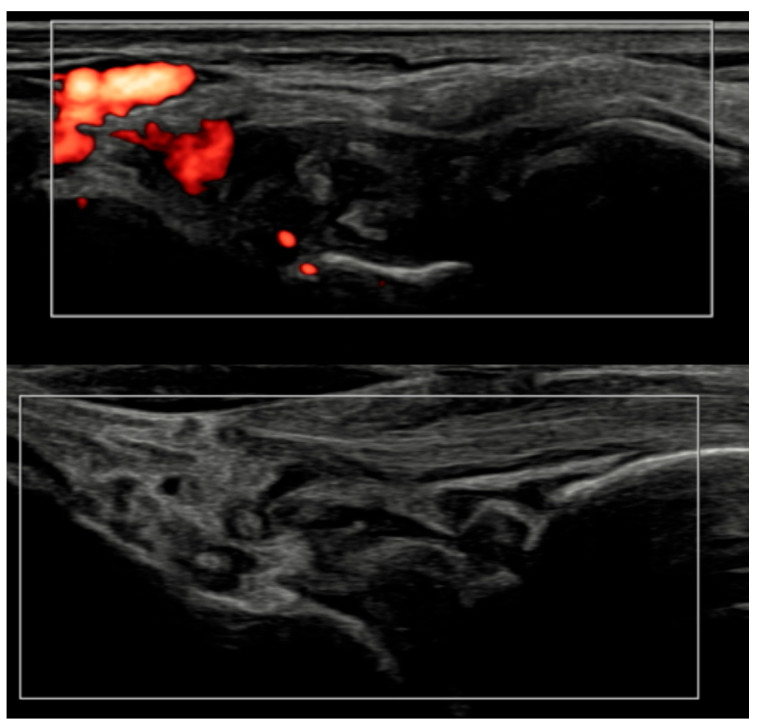
Longitudinal ultrasound comparison of the wrist joint at baseline (T0, **top**) showing synovial hypertrophy (grade 2) with intra-articular power Doppler signal (PD grade 2), corresponding to a combined EULAR–OMERACT synovitis grade = 2 (joint-level), and at 6 months (T6, **bottom**), demonstrating near-complete resolution of synovial hypertrophy and power Doppler signal, with a joint-level EULAR–OMERACT synovitis grade = 0.

**Figure 7:**
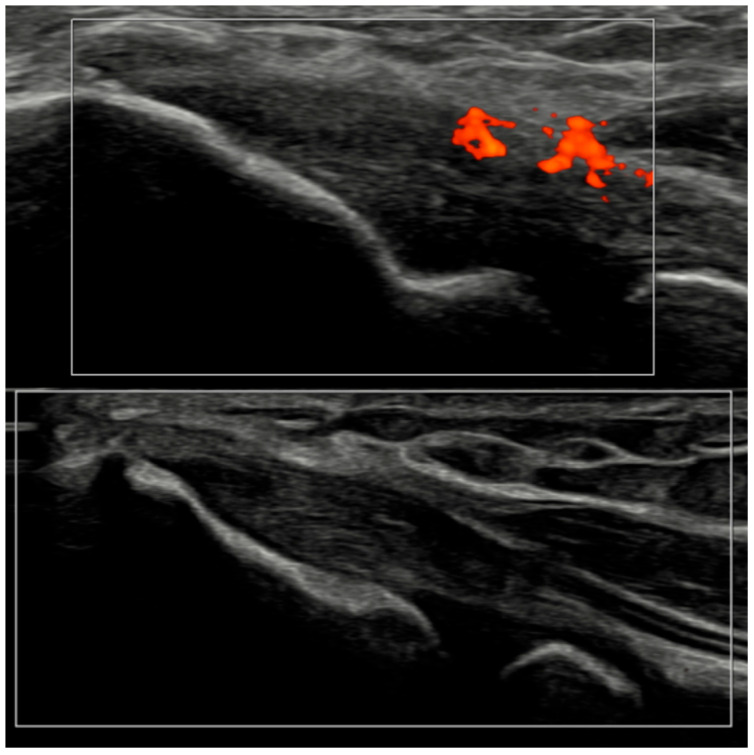
Ultrasound images of the elbow extensor enthesis at baseline (T0, **top** image) showing power Doppler signal (PD grade 2), and at 3 months (T3, **bottom** image), demonstrating resolution of power Doppler signal (PD grade 0).

Several methodological and technical features add credibility to these results. All ultrasound acquisitions were performed on calibrated high-frequency systems using low-PRF Doppler settings per EULAR standards, minimizing artifacts such as anisotropy or compression-induced flow suppression [3]. The synovio-entheseal complex model of PsA pathogenesis provides a conceptual basis for our findings: the anatomical continuity of synovitis, tenosynovitis, and enthesitis explains why vascular suppression under IL-23 blockade manifests coherently across tissues. Representative ultrasound images (Figure 4, Figure 5, Figure 6 and Figure 7) illustrate this evolution at prototypical sites such as the wrist and elbow extensor enthesis. However, mini-entheses of the hand and nail unit were not systematically evaluated; future guselkumab imaging cohorts should incorporate these distal targets, given their diagnostic and prognostic specificity [3].

In routine-care practice, treatment adjustments provide additional context. After the Month 3 evaluation, guselkumab dosing was intensified to q4w in patients due to persistent disease activity, while the remaining patients continued q8w. Because intensification was not randomized and is confounded by indication, these data cannot inform comparative efficacy but illustrate how PDUS information may support individualized decisions. Importantly, the consistency of imaging improvement despite dose heterogeneity indicates a robust biological signal across standard dosing intervals.

This study has strengths and limitations. Its strengths include consecutive recruitment, standardized imaging, single-platform acquisition, and full six-month retention, eliminating attrition bias. Its limitations include the single-center retrospective design, modest sample size, and absence of a control arm. Certain baseline denominators were incomplete owing to routine-care documentation. Operator dependency remains intrinsic to ultrasound, despite consensus scoring and calibration. Furthermore, the observed PD-leading and dispersion trends were exploratory and lacked confirmatory testing. Future multicenter prospective studies should address these limitations and include standardized nail/mini-enthesis protocols to capture PsA-specific microdomains.

Clinically, PDUS provides objective confirmation of biological response to IL-23p19 inhibition, demonstrating reduced Doppler-detected inflammatory activity as early as three months and consolidated improvement by six. While these data should not be interpreted as practice-directive evidence, they support the potential role of PDUS as a hypothesis-generating biomarker in imaging-informed care. Persistent PD-positive GLOESS or OMERACT-2 scores at 3–6 months may signal ongoing inflammation warranting therapeutic review, whereas PD-negative but symptomatic patients may benefit from non-inflammatory pain management strategies. Integrating such imaging checkpoints into prospective treat-to-target frameworks could refine the stratification of responders and non-responders. Ultimately, PDUS complements, not replaces, composite clinical indices by adding a biological dimension to disease monitoring [4,11,13,21,22,23].

In short, our fully ascertained, 20-patient, real-world cohort demonstrates that guselkumab is associated with an early and significant reduction in PD-inclusive ultrasound synovitis by 3 months, with further improvement by 6 months, paralleled by clinical gains across PsA domains. These imaging kinetics are concordant with the time course and durability of improvements reported in DISCOVER-1/2 and extend them by confirming tissue-level vascular quiescence under IL-23p19 blockade [8,9,14,21,24,25,26,27]. The findings also resonate with the ultrasound-anchored ULTIMATE data under IL-17A inhibition, reinforcing PDUS as a sensitive modality for early response detection [7,15]. Together with real-world effectiveness and persistence studies [13,17], our results support the incorporation of PDUS into treat-to-target algorithms at 3–6 months, using PD-inclusive metrics (GLOESS; OMERACT-2) to distinguish true inflammatory non-response from non-inflammatory pain and to guide timely intensification or switching. Finally, our data highlight the added value of PDUS in PsA, beyond clinical indices alone, for confirming biological response, reducing diagnostic uncertainty around enthesitis, and advancing pragmatic, imaging-informed care [2,3,4,12,16,18,28,29].

## 5. Conclusions

In this single-center real-world cohort, guselkumab was associated with significant improvement in PD-inclusive ultrasound synovitis (GLOESS) by 3 months, with further improvement by 6 months. OMERACT entheseal scores also improved, with changes in the activity + structure construct primarily driven by a reduction in inflammatory activity; entheseal structural burden appeared stable over the 6-month interval. Clinical measures (DAPSA, PASI) improved in parallel, and treatment persistence was high at 6 months. These findings are hypothesis-generating and support further prospective controlled studies evaluating standardized PDUS outcomes and longer-term structural trajectories.

## Figures and Tables

**Table 1:** Continuous variables.

Variable	Mean	SD	Median	IQR
Age (years)	58.26	9.45	61	15
Weight (kg)	79.1	16.44	76.5	27.25
Height (cm)	168.4	8.95	165.5	11.75
BMI (kg/m^2^)	27.7	4.21	27.7	5.68
VAS pain (cm)	6.39	2.08	6	3
PGA (cm)	6.23	1.85	6	2
ESR (VES)	26.38	12.16	22	23
CRP (mg/dL)	4.15	2.41	4	3.15
DAPSA (T0)	27.47	5.42	26.95	8.53
PASI (T0)	9.88	11.63	5	18.45
SPARCC	5.10	2.60	4.0	3.0
GLOESS T0	28.5	5.3	28	8.25
OMERACT-1	6.42	2.51	6	3
OMERACT-2	2.88	2.24	3	3

SD, standard deviation; IQR, interquartile range; ESR (VES), erythrocyte sedimentation rate; CRP, C-reactive protein; VAS, visual analogue scale; PGA, patient global assessment; DAPSA, Disease Activity Index for Psoriatic Arthritis; PASI, Psoriasis Area and Severity Index; SPARCC, SpondyloArthritis Research Consortium of Canada enthesitis index; GLOESS, EULAR–OMERACT Global Synovitis Score; OMERACT-1, entheseal score (activity + structure); OMERACT-2, entheseal score (activity-only). *n* = 20.

**Table 2:** GLOESS (24-joint) over time statistics and paired change tests.

Metric	*n*	Mean	SD	Median	IQR	*p*-Value (Wilcoxon)
T0 (baseline)	20	28.5	5.3	28	8.25	—
T3 (Month 3)		24.45	5.39	24.5	9.5	—
T6 (Month 6)		22.8	5.98	23	7.75	—
ΔT0 → T3		−4.05	2.78	−3.50	3	0.0312
ΔT0 → T6		−5.70	4.05	−4.50	5.75	0.0001

GLOESS = EULAR–OMERACT Global Synovitis Score (24-joint composite, higher = more synovitis); ΔT0 → T3/ΔT0 → T6 = within-patient change from baseline (T0) to Month 3 (T3)/Month 6 (T6); *n* = number of non-missing observations contributing to each row; SD = standard deviation; IQR = interquartile range. Within-patient comparisons used two-sided Wilcoxon signed-rank tests.

**Table 3:** OMERACT enthesitis scores over time.

Metric	Mean	SD	Median	IQR
OMERACT-1 (activity + structure)				
T0	8.88	3.23	8.0	2.50
T3	6.88	2.30	7.5	2.25
T6	5.29	2.56	6.0	3.00
OMERACT-2 (activity-only)				
T0	5.38	2.92	4.5	2.25
T3	3.13	1.55	3.0	2.00
T6	1.71	1.70	2.0	1.50

OMERACT-1 = enthesitis score including activity and structural lesions; OMERACT-2 = activity-only definition. T0/T3/T6 = baseline/3-month/6-month visits. IQR = interquartile range; SD, standard deviation.

## Data Availability

The data presented in this study are not publicly available due to privacy and ethical restrictions related to patient confidentiality. Data may be made available from the corresponding author upon reasonable request, subject to institutional approval.

## References

[1] Coates L.C., Helliwell P.S. (2017). Psoriatic arthritis: State of the art review. Clin. Med..

[2] Balint P.V., Terslev L., Aegerter P., Bruyn G.A.W., Chary-Valckenaere I., Gandjbakhch F., Iagnocco A., Jousse-Joulin S., Möller I., Naredo E. (2018). Reliability of a consensus-based ultrasound definition and scoring for enthesitis in spondyloarthritis and psoriatic arthritis: An OMERACT US initiative. Ann. Rheum. Dis..

[3] Bonfiglioli K.R., Lopes F.O.A., Figueiredo L.Q., Ferrari L.F.F., Guedes L. (2024). Ultrasonographic Insights into Peripheral Psoriatic Arthritis: Updates in Diagnosis and Monitoring. J. Pers. Med..

[4] Agache M., Popescu C.C., Enache L., Mogoșan C., Filippucci E., Codreanu C. (2024). Additional Value of Ultrasound in Patients with Psoriatic Arthritis Within Treatment Target. J. Clin. Med..

[5] Freeston J.E., Coates L.C., Nam J.L., Moverley A.R., Hensor E.M., Wakefield R.J., Emery P., Helliwell P.S., Conaghan P.G. (2014). Is there subclinical synovitis in early psoriatic arthritis? A clinical comparison with gray-scale and power Doppler ultrasound. Arthritis Care Res..

[6] Tang T., Jin H., Yang Y. (2024). Ability of the European League Against Rheumatism-Outcomes Measures in Rheumatology combined scoring system for grading dorsal joint space synovitis to accurately evaluate ultrasound-detected hand synovitis. Quant. Imaging Med. Surg..

[7] D’Agostino M.A., Schett G., López-Rdz A., Šenolt L., Fazekas K., Burgos-Vargas R., Maldonado-Cocco J., Naredo E., Carron P., Duggan A.M. (2022). Response to secukinumab on synovitis using Power Doppler ultrasound in psoriatic arthritis: 12-week results from a phase III study, ULTIMATE. Rheumatology.

[8] Deodhar A., Helliwell P.S., Boehncke W.H., Kollmeier A.P., Hsia E.C., Subramanian R.A., Xu X.L., Sheng S., Agarwal P., Zhou B. (2020). Guselkumab in patients with active psoriatic arthritis who were biologic-naive or had previously received TNFα inhibitor treatment (DISCOVER-1): A double-blind, randomised, placebo-controlled phase 3 trial. Lancet.

[9] McInnes I.B., Rahman P., Gottlieb A.B., Hsia E.C., Kollmeier A.P., Chakravarty S.D., Xu X.L., Subramanian R.A., Agarwal P., Sheng S. (2021). Efficacy and Safety of Guselkumab, an Interleukin-23p19-Specific Monoclonal Antibody, Through One Year in Biologic-Naive Patients with Psoriatic Arthritis. Arthritis Rheumatol..

[10] Coates L.C., Ritchlin C.T., Gossec L., Helliwell P.S., Rahman P., Kollmeier A.P., Xu X.L., Shawi M., Karyekar C.S., Contré C. (2023). Guselkumab provides sustained domain-specific and comprehensive efficacy using composite indices in patients with active psoriatic arthritis. Rheumatology.

[11] Din S.U., Saeed M.A., Hameed M.R., Aamer M., Arshad U., Qamar H.Y. (2023). Implementation of the Treat-to-Target Approach in Psoriatic Arthritis and Its Outcomes in Routine Clinical Practice. Cureus.

[12] Granados R.E.M., Ladehesa-Pineda M.L., Puche-Larrubia M.Á., Escudero-Contreras A., Dougados M., Collantes-Estevez E., López-Medina C. (2023). Enthesitis indices identify different patients with this characteristic in axial and peripheral spondyloarthritis and also in psoriatic arthritis: ASAS-PerSpA data. Arthritis Res. Ther..

[13] Mease P.J., Ogdie A., Tesser J., Shiff N.J., Lin I., Chakravarty S.D., Kelleman M., Dodge R., McLean R.R., Broadwell A. (2023). Six-Month Persistence and Multi-domain Effectiveness of Guselkumab in Adults with Psoriatic Arthritis: Real-World Data from the CorEvitas Psoriatic Arthritis/Spondyloarthritis Registry. Rheumatol. Ther..

[14] McInnes I.B., Rahman P., Gottlieb A.B., Hsia E.C., Kollmeier A.P., Xu X.L., Jiang Y., Sheng S., Shawi M., Chakravarty S.D. (2022). Long-Term Efficacy and Safety of Guselkumab, a Monoclonal Antibody Specific to the p19 Subunit of Interleukin-23, Through Two Years: Results From a Phase III, Randomized, Double-Blind, Placebo-Controlled Study Conducted in Biologic-Naive Patients with Active Psoriatic Arthritis. Arthritis Rheumatol..

[15] D’Agostino M.A., Carron P., Gaillez C., Conaghan P.G., Naredo E., López-Rdz A., Šenolt L., Burgos-Vargas R., Hanova P., Padovano I. (2023). Effects of secukinumab on synovitis and enthesitis in patients with psoriatic arthritis: 52-week clinical and ultrasound results from the randomised, double-blind ULTIMATE trial with open label extension. Semin. Arthritis Rheum..

[16] Tan Y.K., Thumboo J. (2025). The EULAR-OMERACT joint-level scoring of ultrasound synovitis demonstrates good construct validity when tested at the patient-level in comparison with measures of disease activity and joint damage in patients with rheumatoid arthritis. Front. Med..

[17] Walsh J.A., Lin I., Zhao R., Shiff N.J., Morrison L., Emond B., Yu L.H., Schwartzbein S., Lefebvre P., Pilon D. (2024). Comparison of Real-World On-Label Treatment Persistence in Patients with Psoriatic Arthritis Receiving Guselkumab Versus Subcutaneous Tumor Necrosis Factor Inhibitors. Drugs Real World Outcomes.

[18] Di Matteo A., Cipolletta E., Destro Castaniti G.M., Smerilli G., Airoldi C., Aydin S.Z., Becciolini A., Bonfiglioli K., Bruns A., Carrara G. (2022). Reliability assessment of the definition of ultrasound enthesitis in SpA: Results of a large, multicentre, international, web-based study. Rheumatology.

[19] Conaghan P.G., D’agostino M.A., Boers M., Naredo E., Mandl P., Carron P., Backhaus M., Lopez-Rodriguez A., Hanova P., Goyanka P. (2023). E087 Reduced joint synovitis assessment versus the global EULAR OMERACT synovitis score (GLOESS) to predict the response to secukinumab in patients with active psoriatic arthritis and inadequate response to conventional disease-modifying anti-rheumatic drugs: Exploratory results from the ULTIMATE trial. Rheumatology.

[20] D’Agostino M.A., Schett G., Rodríguez A.L., Šenolt L., Maldonado-Cocco J., Burgos-Vargas R., Naredo E., Carron P., Boers M., Duggan A.M. (2021). P187 Secukinumab significantly decreased joint synovitis measured by Power Doppler ultrasonography in biologic-naive patients with active psoriatic arthritis: Primary (12week) results from a randomised, placebo-controlled Phase 3 study. Rheumatology.

[21] Ritchlin C.T., Deodhar A., Boehncke W.H., Soriano E.R., Kollmeier A.P., Xu X.L., Zazzetti F., Shawi M., Jiang Y., Sheng S. (2023). Multidomain efficacy and safety of guselkumab through 1 year in patients with active psoriatic arthritis with and without prior tumor necrosis factor inhibitor experience: Analysis of the phase 3, randomized, placebo-controlled DISCOVER-1 study. ACR Open Rheumatol..

[22] Wervers K., Vis M., Tchetveriko I., Gerards A.H., Kok M.R., Appels C.W.Y., van der Graaff W.L., van Groenendael J.H.L.M., Korswagen L., Dieren J.J.V. (2018). Burden of psoriatic arthritis according to different definitions of disease activity: Comparing minimal disease activity and the disease activity index for psoriatic arthritis. Arthritis Care Res..

[23] Ritchlin C.T., Mease P.J., Boehncke W.H., Tesser J., Chakravarty S.D., Rampakakis E., Shawi M., Schiopu E., Merola J.F., McInnes I.B. (2024). Durable control of psoriatic arthritis with guselkumab across domains and patient characteristics: Post hoc analysis of a phase 3 study. Clin. Rheumatol..

[24] Zhao C., Zhuang N., Zhang Y., Lv H., Zhang W., Shen Y., Wu W., Tian Y., Xie L., Zhou G. (2025). Reliability and Availability of the 2017 EULAR-OMERACT Scoring System for Ultrasound Synovitis Assessment: Results from a Training and Reading Exercise. J. Ultrasound Med..

[25] D’Agostino M.A., Boers M., Gaillez C., Gamez C., Ventura L., Rosa J., Padovano I., Mandl P., Kleyer A., Bakewell C. (2022). *Op*0260 responsiveness of a combined power doppler and greyscale ultrasound score for assessing synovitis at joint level in psoriatic arthritis patients with inadequate response to csdmards: Data from the ultimate trial. Ann. Rheum. Dis..

[26] McGonagle D., McInnes I.B., Deodhar A., Schett G., Shawi M., Chakravarty S.D., Kollmeier A.P., Xu X.L., Sheng S., Xu S. (2023). Guselkumab, a Selective Interleukin-23 p19 Subunit Inhibitor, Resolves Dactylitis in Patients with Active Psoriatic Arthritis: Pooled Results Through Week 52 From Two Phase 3 Studies. ACR Open Rheumatol..

[27] Mease P.J., O’Brien J., Middaugh N., Kricorian G., Stryker S., Collier D.H., Ogdie A. (2023). Real-World Evidence Assessing Psoriatic Arthritis by Disease Domain: An Evaluation of the CorEvitas Psoriatic Arthritis/Spondyloarthritis Registry. ACR Open Rheumatol..

[28] Filippucci E., Smerilli G., Di Matteo A., Grassi W. (2021). Ultrasound definition of enthesitis in spondyloarthritis and psoriatic arthritis: Arrival or starting point?. Ann. Rheum. Dis..

[29] Mease P.J., Ogdie A., Tesser J., Shiff N.J., Zhao R.S., Chakravarty S.D., Kelleman M., Dodge R., McLean R.R., Broadwell A. (2024). Improvements in Patient-Reported Outcomes Through Six Months of Guselkumab Treatment in Patients with Active Psoriatic Arthritis: Real-World Data from the CorEvitas Psoriatic Arthritis/Spondyloarthritis Registry. ACR Open Rheumatol..

